# Social identity loss and reverse culture shock: Experiences of international students in China during the COVID-19 pandemic

**DOI:** 10.3389/fpsyg.2023.994411

**Published:** 2023-02-09

**Authors:** Rameez Raja, Jianfu Ma, Miwei Zhang, Xi Yuan Li, Nayef Shabbab Almutairi, Aeshah Hamdan Almutairi

**Affiliations:** ^1^Department of Sociology, School of Sociology and Anthropology, Sun Yat-sen University, Guangzhou, China; ^2^Pakistan Studies Centre, North Minzu University, Yinchuan, Ningxia, China; ^3^PKUFH-Ningxia Women Children’ Hospital, Yinchuan, Ningxia, China; ^4^Department of Public Health, Al-lith College of Health Sciences, Umm Al-Qura University, Mecca, Saudi Arabia

**Keywords:** international students, the COVID-19 pandemic, reverse cultural shock, internationalization of higher education, life transition, China, social identities, mindsponge mechanism

## Abstract

**Background:**

International students are often exposed to various challenges during life transitions. The ‘mindsponge’ mechanism suggests that individuals absorb and integrate new cultural values that are compatible with their core values while rejecting those of lesser importance. On the basis of this notion, this article explores the experiences of international students in China regarding their unplanned return to their home countries during the COVID-19 pandemic through the lens of the mindsponge mechanism.

**Aim:**

This article aims to highlight the experiences of international students in China who are going through life transitions due to the global pandemic. The study focuses on the experiences of two groups of international students: (1) Those who remained in China during the pandemic, and (2) those who had left China and were stranded in their home countries due to a ban on international travel amid COVID-19.

**Method:**

This qualitative study comprised of in-depth semi structured in-person and online interviews. Thematic Analysis was used to analyze the data in order to generate study themes.

**Results:**

The results revealed that students who remained in China experienced challenges which included anxiety, closure of campuses, lockdown, their parents’ concern regarding health issues, and not being able to meet with friends. On the other hand, students who had left China during the pandemic were confined to their home countries. This group of students experienced more severe problems than the students who remained in China. Since the transition to home countries was “unplanned,” they were not ready to readjust to their native culture and were vulnerable to severe reverse culture shock. Upon returning to their home countries, international students faced a number of challenges, including readjustment to their home countries and changes in their lives in host and home countries. In addition, they lost social and academic resources, such as the disruption of study environment, losing important group memberships, financial constraints, visa expiry, graduation delay, and academic suspension.

**Conclusion:**

This study concluded that the international students experienced cultural problems after unplanned transition to their home countries during the pandemic. They described effects of reverse culture shock as being more distressing. They perceived dissatisfaction due to loss of previously held social identities and sense of belonging to the traditional society they left behind. There is a need of future studies on the long-term effect of unplanned transition on psychological, social and professional experiences. The process of readjustment has proven to be a challenging endeavor.

## 1. Introduction

Globalization has produced numerous changes making the world like a global village. As a result, students often choose to study abroad not only to gain quality education, but also to aquire multicultural characteristics (Cruwys et al., 2015; Ng et al., 2018). Many parents consider international education necessary for their children to be availed of opportunities in multinational corporations. International students possess unique characteristics due to diverse group membership while studying overseas making them different from local students (Ng et al., 2018; Raja et al., 2021).

Apart from many advantages of international education, international students often confront perceived challenges before, during, and after the application process that are quite different from domestic students’ experiences. They must be well prepared to apply by taking numerous exams, apply to the intended program by sending documents overseas, and waiting for the institution’s response after their application causes international students to experience stress even before their classes start. However, even after successfully applying, studying abroad is not the end of the story for international students. It is just the beginning of a new story about their academic and social adjustment. Life transition involves challenges that impact peoples’ well-being in a new society. Even if a transition is positive, it affects individuals (Miller, 2010) as it challenges the sense of self-continuity and associated social identities (Haslam et al., 2008). Many transitions are positive where individuals enhance their self-esteem and efficacy (Haslam et al., 2020). However, in the transition phase, international students struggle to maintain and gain social networks for successful adjustment and well-being (Ward et al., 2020).

Therefore, accepting a cross-cultural mindset is likely to be a prerequisite for international students’ successful adjustment and well-being during the transitory phase. There is a notion that describes how a person’s mindset integrates new values and ejects unimportant onestherefore, the current study raises the question of how international students in China have been affected mentally due to the unplanned transition back to their home countries amid the COVID-19 pandemic. With the “mindsponge” theory (Vuong and Napier, 2015) this study tries to explain why and how international students could adjust to their native cultural values on their unplanned return to home countries during the pandemic. The article attempts to highlight the re-accultrative stress faced by international students who have been successfully adjusted in China during their studies. Accordingly, the study demarcates that the shift of “mindset” of international students can be explained through a “mindsponge process,” where the personal identity such as “Who am I?” to “How I should be?” Therefore, the present study aims to understand how life-changes impact international students’ sense of belonging amid the COVID-19 pandemic, focusing on the dimensions of identity through the mindsponge mechanism.

### 1.1. International students in China and the COVID-19 pandemic

According to the Ministry of Education, China, 492,185 international students from 196 countries were enrolled in various universities and Higher Educational Institutes (HEIs) excluding Hong Kong, Macau, and Taiwan (MoE, 2020). While Chinese universities were enrolling a growing number of international students, the pandemic disrupted educational activities, putting pressure on the internationalization of higher education and students (Yang, 2020). COVID-19 was identified in mid-December 2019 in Wuhan, China, and later it spread rapidly to other cities due to massive human mobility across cities during the Chinese New Year (Peters et al., 2020). Initially, people were unaware of the novel virus and were fearlessly roaming across the country. Meanwhile, the news was circulated about the disease while people were busy with New Year preparations. Many international students traveled to their home countries for holidays, while some were on campus during spring holidays, especially those engaged with their research work or dissertation.

Suddenly, an increasing number of cases were reported, and the government imposed a lockdown to minimize the spread of the virus. According to World Health Organization, COVID-19 has affected all age groups, sexes, ethnicities, and social classes (WHO, 2020). The pandemic disrupted educational activities, travel, and other social aspects for local students and for international students in particular. It also compelled Chinese universities to cease further enrollment which reduced the pace of the internationalization of higher education (Yang, 2020). Several international students who had left China for the holidays were stranded in their countries, struggling to return to China. Those students who were already in China during the pandemic could witness new experiences in how China dealt with the pandemic. Although the pandemic affected international students psychologically, socially, academically, and financially due to their immigrant status (Bozkurt et al., 2020), the experiences of international students in China did not receive due attention in the literature. Therefore this article tries to fill this gap and add knowledge about the Chinese context into the literature.

## 2. Literature review

Global pandemic remarkably affected international students’ mobility that has direct impact on internationalization of higher education (Altbach and De Wit, 2020; Mok, 2020). Many students across the globe shifted their mind regarding study overseas due to the travel restriction and closure of higher educational institutes. For instance, a significant decline in international students enrollment has been recorded in top destinations such as the United States, United Kingdom, and Australia. According to the Institute of International Education (IIE) survey, colleges and universities in the US estimated an approximate 90% decrease, and higher educational institutes experienced a 30% decrease in international students’ enrolment for the academic session 2020–2021 (Martel, 2020). Likewise, 39% of Chinese students in the United Kingdom were uncertain regarding their study plans, resulting in a significant decline in United Kingdom higher educational institutes (Durnin, 2020). Similarly, enrollment of Chinese students were also declined in Australia which affected the international education industry (Mercado, 2020).

Higher education institutes have experienced substantial challenges such as switching to online lectures/tutorials, closing libraries, changing contact channels for instructors and administrative support, new evaluation methods, varying workloads and performance levels (Bezerra, 2020; Cao et al., 2020; Gallego et al., 2020; Kamarianos et al., 2020; Khan, 2020; Owusu-Fordjour et al., 2020). Furthermore, the pandemic also presented social challenges to international students such as no meetings with friends, university colleagues, or relatives, no parties, no traveling, and stranded in their home countries. In addition, students faced financial impacts such as loss of student jobs, fear about their future education and career (Brooks et al., 2020; Elmer et al., 2020).

On the other hand, online learning played a vital role in distant learning during the pandemic. However, several studies highlighted that students and teachers have expressed unsatisfactory concerns regarding online education for effectiveness in learning and interaction (Händel et al., 2020; Herman, 2020; Xiong et al., 2020). In addition, research based students who required to work in labs or fields remained the most vulnerable group during the pandemic and their experiences did not receive proper attention. Existing literature mainly focused on international students who were unable to return to their home countries and experienced academic, social, and financial challenges (Bilecen, 2020; Jenei et al., 2020; Zhai and Du, 2020; Hari et al., 2021). Studies mostly focused on academic work and students’ mental health (Huckins et al., 2020; Kaparounaki et al., 2020; Sahu, 2020; Tang et al., 2020; Zimmermann et al., 2020). A few studies explored other aspects of the pandemic, including its impact on the economy, society, and the environment. There is a limited knowledge regarding the impact of pandemic on international students who have been stranded in their home countries. Few studies, although, highlighted the experiences of international students studying from home countries during the pandemic (Wilczewski et al., 2021; Dong and Ishige, 2022). However, these studies emphasized mainly on online learning from home countries and draw comparison between international students in host and home countries. In addition, these studies have not captured the experiences of research based international students who had no access to labs, fieldwork, and research related activities. Existing studies showed findings from the early stages of the pandemic with a narrow focus on experiences of international students stranded in home countries. Therefore, the current study tries to unfold the factors impacting international students’ social identity shift and re-acculturative stress in home countries due to unplanned transition during the pandemic.

### 2.1. Culture shock and reverse culture shock

Previous studies have shown that international students frequently experience culture shock when they first travel to and reside in a new country (Hendrickson et al., 2011; Hotta and Ting-Toomey, 2013). The process of transition to a new cultural context is described as “culture shock” (Pedersen, 1994). According to Zhou et al. (2008) emotional, psychological, and/or physical problems are associated with culture shock which can minimize or prolong an individual’s adaptation period to a new cultural milieu (Ward et al., 2001). Therefore, the international students passes through a transitional period for adjusting to the host culture along with getting pace with their academic requirements. Sometimes, the stability and equilibrium gained after a certain period is shattered due to some forceful events leading these students to return to their home countries. Such students after returning to home countries often face another form of cultural shock which is termed as reverse culture shock.

Reverse culture shock emphasized on the socio-psychological challenges of returning to home culture after sojourning in another cultural milieu (Gaw, 2000). After adapting to the host country, they must go through a period of readjustment in their native culture. Under normal circumstances, international students after completing their targeted educational tenure can easily phase out the reverse culture shock. However, due to multiple challenges in home countries, unplanned transitions during the educational tenure make international students particularly vulnerable to reverse culture shock. Since both reverse culture shock and culture shock have an impact on adjustment, very little is known about reverse culture shock in the context of “unplanned or forced return to one’s own culture.” In particular, there is limited knowledge about how the reverse culture shock affects already held social identities in host country. After spending a long time abroad, some researchers have investigated the process of experiencing reverse culture shock on returning to one’s home culture (Xia, 2009; Pritchard, 2011; Kartoshkina, 2015; Yang et al., 2018). Previous literature have shown that reverse culture shock is more problematic and harmful than culture shock (Xia, 2009). The home returnees face a variety of psychological difficulties, many of which are related to their relationships with family and friends. Fears, phobias, feelings of isolation and rejection, anxiety and sadness, are common experiences (Le and LaCost, 2017; Winkel et al., 2021). Therefore, this study specifically highlights the experiences of international students who returned their home countries and experienced reverse culture shock as a result of social identity shift through mindsponge mechanism.

In specific, the study highlights two groups of international students; (1) those who remained in China during the pandemic, and (2) those who have left China and have been stranded at home countries and unable to return to their host country. This study will enhance our understanding of the student’s experiences in both scenarios, i.e., being in the host or home countries and will provide novel support to the international students studying abroad literature.

## 3. Theoretical framework

Individuals possess a mindset with some significant cultural norms and values which are the part of their identity (Correll et al., 2004). Through these cultural values, individuals review the significance of information and respond accordingly. The blue circle indicates comfort zone of mindset which filter the information and safeguard the core values. During the filtering process, information is scrutinized and compared with core values where some compatible information are integrated while some are rejected (Levy et al., 2007; Vuong and Napier, 2014). The yellow space shows peripheral values which needs to be undergo scrutiny before integrating or rejecting (Vuong and Napier, 2014). The filtering process consists of three factors: (1) assessment of value for appropriateness, (2) evaluation for new insights, and (3) judging whether the values provides new ideas and opportunity (Napier and Nilsson, 2008; Napier and Hoang Vuong, 2013; Vuong and Napier, 2015).

Through the mindsponge mechanism, the study unfolds experiences of international students regarding their unplanned (forced) return to home countries due to the pandemic. It describes that during the pandemic, the change in international students’ value system is due to information filtering—the decision to either accept or reject newly acquired information & values. The process updates international students’ identity, attitudes, and behaviors. While the goal is to adapt to the current living environment, the decision of whether to accept or reject a certain value is based on cost–benefit analysis in pandemic contexts. The return of international students to home countries due to the pandemic entailed severe reverse culture shock (Winkel et al., 2021). This is particularly necessary to understand what students encounter during a global pandemic in host countries and how the unplanned life transition to home countries affect the sense of belongings. To adequately address these issues, there is a need to explore the lived experiences of international students in China and those who have been stranded in home countries and unable to return to their host destination due to the pandemic.

## 4. Method

This article is part of the Ph.D. research of the first author. The aim of the study was to investigate the experiences of international students returned to their home countries amid the COVID-19 pandemic. Through mindsponge mechanism, the study employed a qualitative approach to record students’ experiences during the pandemic. The researcher approached international students to participate in study through personal contacts and social media platforms. The snowball method was used to reach the targeted participants who returned to their home countries during the pandemic (Creswell and Poth, 2016). The participants included only those international students who stayed in China for more than 3 years and experienced the pandemic situation. The interviews included diverse international students to ensure impartiality and inclusiveness. The participants were informed that their involvement was voluntary and that the data they provided would not be linked to any personally-identifying information. Participants provided their verbal consent before the interviews. Firsthand information was collected through social media platforms such as WeChat and Twitter. International students often discussed the pandemic and its effects on their academic and social lives in social media groups.

The international students were asked for their prior consent, and confidentiality was ensured. The researcher conducted in-depth semi structured interviews with 20 participants. Five participants were selected from different universities that remained in China during the pandemic (Table 1). whereas 15 participants were those, who had left China and were stranded in their home countries (Table 2).

**Table 1:** Detail of international students who remained in China during the pandemic.

Respondent#	Gender	Nationality	Degree	First arrival in China
01	Male	Kenya	Masters	September 2018
02	Male	Pakistan	Ph.D.	September 2016
03	Female	Iran	Ph.D.	September 2015
04	Female	Turkey	Ph.D.	September 2016
05	Male	Thailand	Ph.D.	September 2018

**Table 2:** Demographic characteristics of the respondents who had left China during the pandemic.

Respondent#	Gender	Nationality	Degree	First arrival in China
01	Male	Pakistan	Ph.D.	September 2018
02	Male	Bangladesh	Ph.D.	September 2017
03	Male	Pakistan	Ph.D.	September 2017
04	Male	India	MBBS	September 2018
05	Female	Thailand	Masters	September 2018
06	Female	Nepal	MBBS	September 2018
07	Female	Latvia	Ph.D.	September 2016
08	Female	Sri Lanka	MBBS	September 2018
09	Female	Vietnam	Ph.D.	September 2017
10	Male	Germany	Masters	September 2018
11	Male	Pakistan	Masters	September 2018
12	Female	India	MBBS	September 2018
13	Male	Vietnam	Ph.D.	September 2016
14	Male	Bangladesh	Masters	September 2018
15	Male	Pakistan	Ph.D.	September 2017

### 4.1. Interview

In-depth semi structured interviews were conducted with the participants during three phases as per the convenience of the participants. First phase was initiated in April 2020 followed by second phase in November 2020. The last phase was held in March 2021. In most cases, face-to-face interviews were not feasible; therefore, online interviews were conducted using social media applications, including WeChat and WhatsApp (Twis et al., 2020). The rapid advancement in communication technologies helped the researchers expand the qualitative data collection through modern communication technologies that provided timely access to the study participants and helped to minimize social desirability bias (Adhabi and Anozie, 2017). The means and channels of data collection, including text and multimedia messages through mobile phones and laptops, have essential contributions in collecting data and ease the research work for researchers (Udtha et al., 2015).

Since the study highlighted lived experiences of international students, the authors applied a phenomenological approach to describe the meaning for students of their lived experiences of a concept or a phenomenon. Phenomenological approach focuses on unfolding what all respondents share in common as they experience a phenomenon such as grief, being left out, or passing through difficult phase of life (Van Manen, 2016). The authors then collected data from the international students and draw description of the spirit of their experiences. The description comprises of “what” the international students experienced and “how” they experienced it (Moustakas, 1994).

Participants were asked about (a) their experiences of leaving China during the pandemic, (b) their current experiences in their home countries, (c) their potential challenges, (d) the social relationships they were involved in, (f) their social connections with other co-national students, local Chinese friends, and multinational peers, and (g) their views about academic and social positioning. The topics were not presented in any particular order and instead arose naturally during the interview. The responses related to myriad challenges brought by the transition due to COVID-19 were focused, along with narratives that speak to social connectedness and isolation.

The length of each interview was 40 min–1 h approximately. Since many participants could communicate in English due to their English academic backgrounds, therefore the interviews were conducted mainly in English. In addition, few interviews were conducted in the participants’ native language (Urdu). The interviews were audio recorded and transcribed in verbatim. The transcription process has been done by the research group of the authors. Essential notes have been taken during the interviews. The transcription sheets were reread and crossed checked by the research group to enhance accuracy.

### 4.2. Analytical strategy

An inductive (data-driven) and deductive (theory-driven) approach was used to conduct a theme analysis. Braun and Clarke (2006, 2012) presented a process for thematic analysis to frame qualitative data in themes forms to capture events and experiences. Coders read transcribed interviews from beginning to end in the first step. Coders labeled introductory sentences in the transcript to identify a feature. This procedure was continued until all phrases were coded and no new theoretically important themes emerged (Braun and Clarke, 2012). The process involved grouping codes that indicated a meaningful pattern in the data (searching for themes phase). Themes were then cross-checked by two researchers and identified codes. The researchers were Ph.D. students and group mates of the first author who assisted in conducting interviews. After thorough deliberation, a few new themes emerged, while others were compressed to produce the study’s main themes, supported by participants’ quotes.

## 5. Findings

### 5.1. International students’ experiences during COVID-19 in China

This section alludes to international students’ experiences and responses who remained in China when the outbreak hit Wuhan and rapidly spread all over China. During the initial phase, the researcher also witnessed and was passing through the same experiences as other international students in China. This was the most crucial period ever experienced by international students because, until this, the virus was found and spread only in China. However, in a month or so, it spread to other countries. The transition from everyday life to lockdown and isolation severely affected the international students’ ability to respond to the situation, putting them in an uncertain state of mind. The families of international students were highly anxious and worried about the health and well-being of their children. During the lockdown, the researcher interviewed one of the Doctoral students who was a student in Wuhan on January 15, 2020, through a WeChat video call. He expressed that “the university was cordoned off, and the students were not allowed to leave their rooms and to meet other students. There was little food stock available due to the lockdown, and the students experienced a food shortage initially. However, after some time, the university started providing necessary food items to each student in the dormitory.” The sudden changes brought by COVID-19 disrupted the routine life of international students, such as their sleep order, the routine of meals, and lack of interest in the study.

As a result of the lockdown, international students were limited to their rooms and remained active only on social media such as WeChat to connect with their friends. All the students were confined to their rooms, and their mobility was limited. On the one hand, social media groups provided an essential channel for connecting with peers, but at the same time, people were sharing videos and information about the COVID-19 affected cases and increasing deaths, which put the students in fear and psychological stress.

These videos and sometimes fake news negatively impacted international students already going through a complex state of mind. Due to a lack of social and emotional support, international students were often experiencing loneliness, stress, and anxiety. When the situation worsened in China and Wuhan in particular, the global media reported the problem in China, which severely put the parents of international students in anxiety and depression. The parents protested in many countries for the safe exit of their children from China.

“My parents were very disturbed when they watched news channels. Many parents of international protested are in front of Foreign offices to send us back home” (Male, Ph.D. student, Pakistan)

In these challenging times, international students could not focus on academic and research work. The research work of postgraduate and doctoral students was severely affected and caused severe delays in experiments that readily involved their upcoming graduation, which was expected in June 2020.

“This outbreak disrupted the progress of my lab work. I couldn't conduct experiments which was the important part of the thesis. I was worried that this may cause a delay in my graduation” (Female, Ph.D. student, Iran)

It is pertinent to mention that a complete lockdown was enforced in Wuhan in January 2020; however, the situation in other cities was not tough. International students in other cities were rushed to book air tickets and leave China. However, with every passing day, the restrictions became more stringent, and several flights were canceled from the first week of February 2020. Most of the students left China in January and the first week of February 2020, and after that, no flight was available due to a ban on international flights.

“We are facing isolation and stress because of COVID-19 and staying in rooms all the time. Therefore, I decided to return home, but my flight was canceled twice. Now, I have booked a connecting flight for February 04, 2020. I hope this time the flight won’t be canceled” (Female, Ph.D. student, Turkey)

This was when COVID-19 was at its peak, and international students tried to go back home so that the lethal virus would not infect them. Many international students in this period left China, and some could not. Various students stuck in Wuhan started an online campaign to highlight their worries and demanded that their governments arrange special flights for them to return to their home countries. This group of international students had been making group videos where they raised issues and problems to return home safely. These students approached their respective embassies in China and informed them about the prevailing scenario. The embassies announced the registration of their students who wanted to return to their countries and made lists. Negotiating with Chinese authorities, some countries have taken their students back from Wuhan through special flights.

“The situation is terrible. We are restricted in our rooms. We requested our Embassy, and they are arranging flights for us to go back home. Our families are also worried about this situation” (Male, Ph.D. Student, Pakistan)

**Figure 1:**
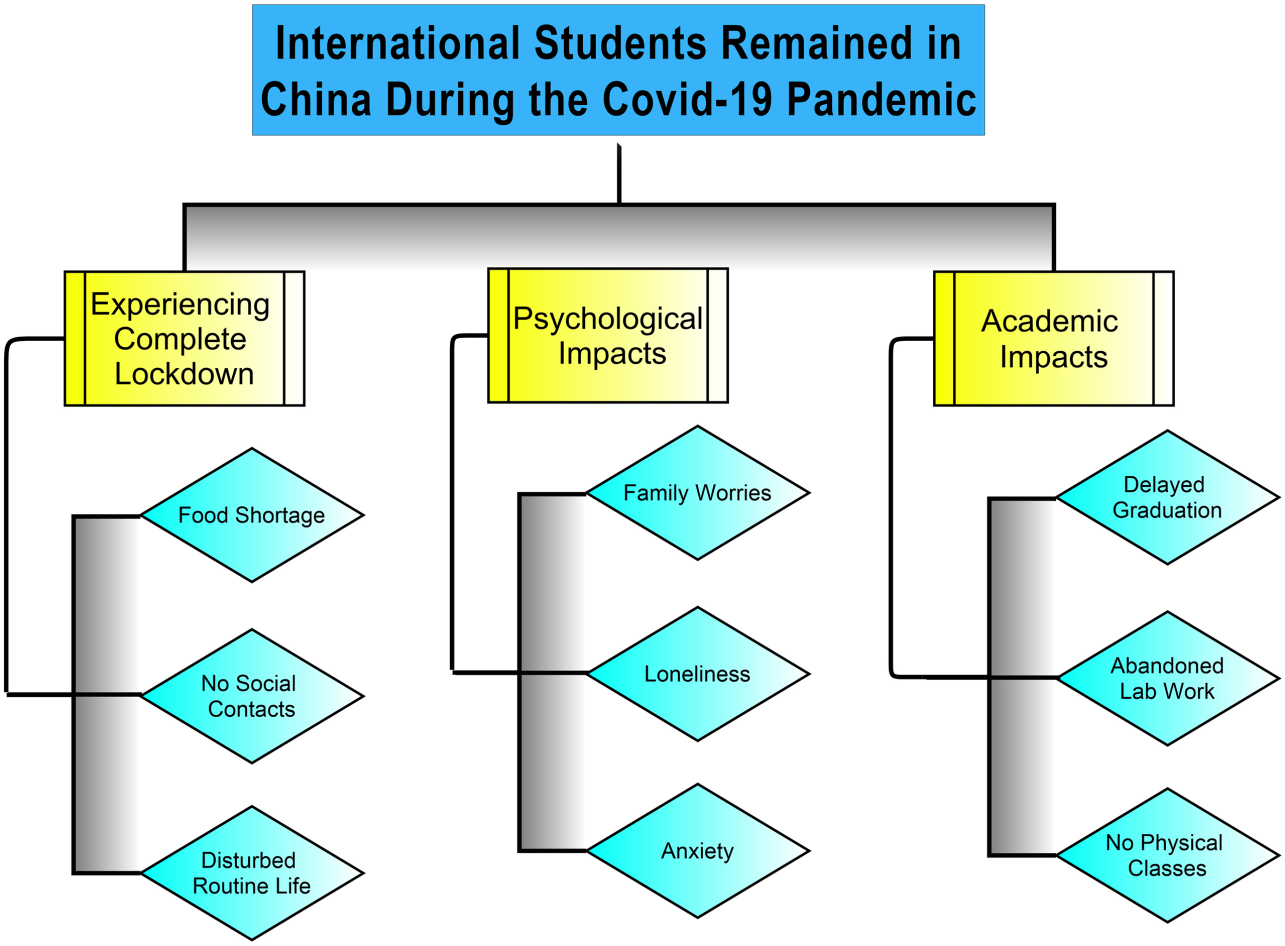
Schematize representation of themes regarding International students who remained in China during the pandemic.

In this theme, the study revealed potential threats to international students’ well-being during the initial phase of COVID-19. Students’ experiences highlighted significant obstacles they faced during their stay in China, such as social immobility, isolation, food shortage, stress, family worries, media hype, cancelation of academic affairs, loss of group attachment, etc. Figure 1 shows a schematic representation of the study themes.

### 5.2. International students who had left China during the pandemic

**Figure 2:**
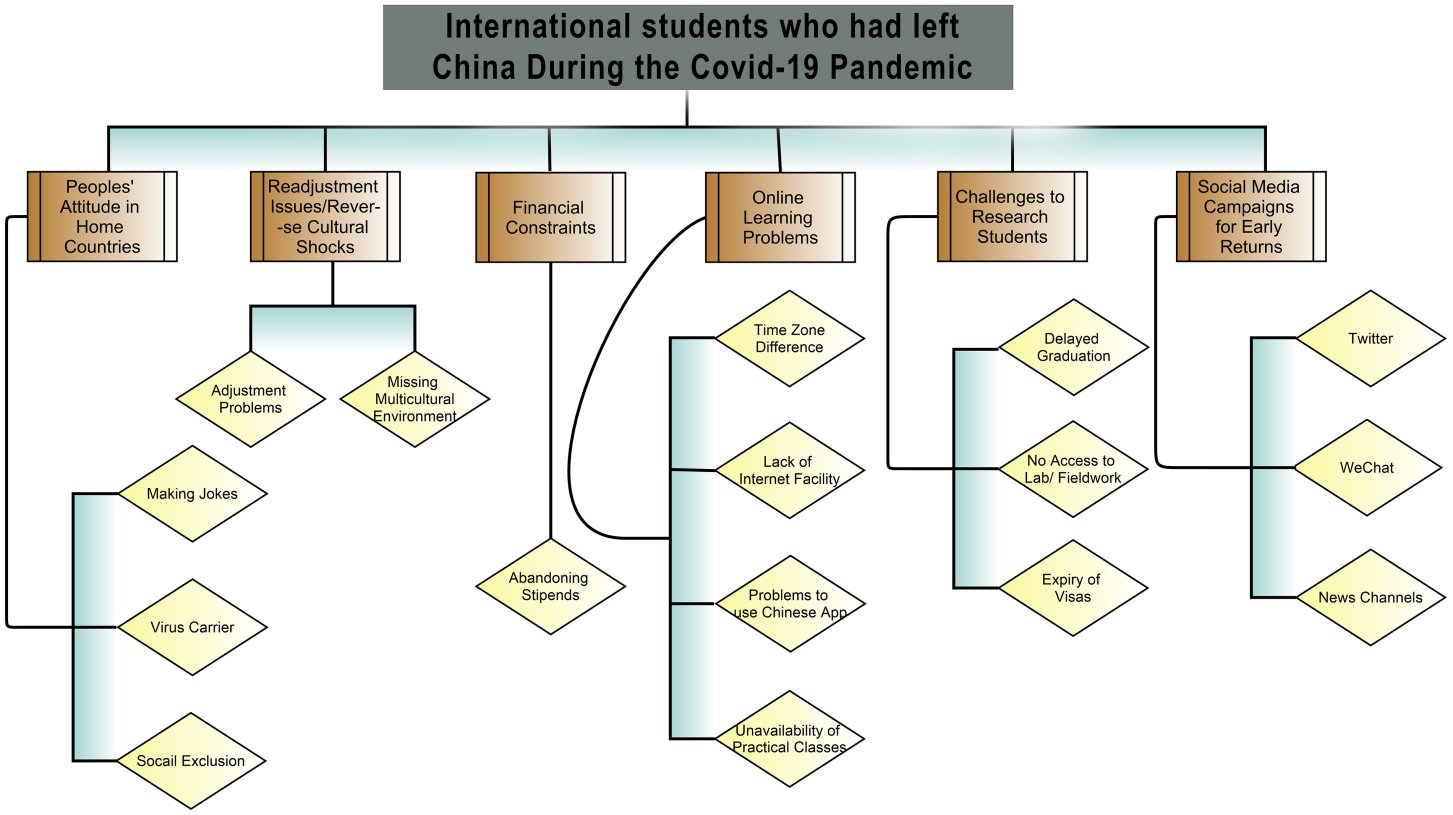
Schematize representation of themes regarding International students who had left China during the pandemic and stranded in their home countries.

The findings constitute the experiences of international students who left China during the pandemic and stranded in their home countries. The data reveals various aspects of international students who were out of China since February 2020 and stranded in their home countries due to a ban on international students’ mobility. It shows potential threats to international students’ well-being as they were distanced from their schools for a long period of time, and their academic career was disrupted by specific issues related to online learning. For instance, postgraduate research students were unable to carry on their lab work and experiments. They faced severe problems that caused stress and anxiety. Many students gave up their studies as they could not make it convenient to complete their thesis requirements. International students encounter financial constraints in paying tuition fee. The scholarship students were also facing financial problems because of not getting their monthly stipends since July 2020. Figure 2 schematically represents the thematic concerns of international students.

#### 5.2.1. Virus carriers

When the international students returned to their home countries, they experienced discomfort and discrimination related to stereotypical assumptions about their exposure to the novel Corona virus. They were stigmatized as “virus carriers.” People used to make jokes of them when they met friends, neighbors, and relatives who taunted them about whether they carried a virus from China. The students initially experienced an unwelcoming attitude in their home countries, and people considered them as coming from the virus center and carriers. Many people avoided meeting the students because they could be infected if they contacted people coming from China.

“When I returned, my friends and other people started joking when they saw me. Some said, ‘do not shake hands; maybe he carried viruses. Some people were reluctant to see me because they truly assumed I was a virus carrier as I came from China recently” (Male, Masters Student, Germany)

“To begin with, people behaved quite weirdly when they saw me. Some ignored me and tried not to meet me because I came from China. This was a bad feeling because I already suffered a lot for coming back home through many airport inspections and other strict immigration processes” (Female, MBBS student, Nepal)

The theme explained how international students were exposed to an unwelcoming attitude from their surroundings upon arrival in their home countries. Even though facing a challenging situation in China during the COVID-19 outbreak, international students also encountered a different attitude from people in their countries which resulted in a great reverse culture shock and a sense of social identity loss.

#### 5.2.2. Readjustment issues

Generally speaking, readjustment issues are faced by most students who return after spending few years abroad because of their studies. But as they know and plan their return to their home countries, they are mentally prepared for such issues and can sustain them effectively. But the case of those students who suffered from the pandemic was even more severe because of the sudden outbreak and their return which was not well planned.

International students encountered a crucial issue related to readjustment in home societies that caused reverse culture shock. The data revealed that international students were possessing diverse social networks and involved in multicultural environment in China. The transition from a multicultural environment to home culture also brought severe readjustment issues in home countries. For instance, participants from developing countries explained that they lived a comfortable life in China. Having international friends and a cross-cultural environment, their confidence and self-esteem were enhanced. The multicultural environment contributed to their freedom and unique skills different from local students.

“Life in China enhanced my confidence by interacting and friendships with students from many countries. Now, I miss all my international friends. We had a perfect time together. We played games, hung out for a meal, and studied together. But now, I think I just woke up from a beautiful dream” (Male, MBBS student, India)

“My life standard in China was high because we had access to many facilities such as 24/7 availability of internet & electricity, well-equipped playgrounds, amusement parks, international gatherings, etc. Which is not available here. For example, I lived in Nanchang, which is not very modern compared to other cities in China like Guangzhou, Beijing, and Shanghai, etc. but still, it is more developed than our capital city (Islamabad)” (Male, Ph.D. student, Pakistan)

The participants highlighted that life in China was exciting, where they could have opportunities to participate in cross-cultural events, such as international cultural days. International students from various countries represent their culture, food stalls, clothes, and cultural music and dance at such events. These events positively contributed to the cross-cultural learning of international students and provided a diverse atmosphere. With the transition from a diverse and multicultural social life, the international students in their countries felt dissatisfaction due to the unavailability of social resources they had while studying in China. This factor pose additional challenges to social identity continuity with old group membership.

In late March and early April 2020, COVID-19 spread worldwide, and the restrictions such as lockdown, social distancing, and a ban on social mobility were enforced by countries which was initially practiced in China. The participants remained more vulnerable to the COVID-19 spikes and being stripped of their international student’s status. During the interviews, the researcher observed that many participants were dissatisfied with leaving China due to COVID-19, because China had successfully controlled the spread of COVID-19 and had taken adequate measures. By May 2020, China had primarily overcome the situation and brought back the routine by easing restrictions.

“Life in China was fantastic. I had many friends. We participated in cultural parties twice a year and learned much about different cultures. I feel that life is boring now, and COVID-19 has changed our lives” (Female, MBBS student, Sri Lanka)

“I think we reacted in a hurry and left China. It was a wrong decision. Now life is normal in China, and we are suffering here. China is not allowing us back now, and our studies and academic period is wasting” (Male, Ph.D. student, Pakistan)

This section highlights the experiences of international students regarding a change in routine life and readjustment issues that resulted in reverse culture shock. Most participants believed they had made a wrong decision by leaving China during the pandemic because China had controlled the situation promptly and effectively.

#### 5.2.3. Financial challenges

As a part of national policy, the Chinese Scholarship Council (CSC) awards various scholarships to attract international students to study in China. The Chinese scholarships include the Chinese Government Scholarship (CGS), MOFCOM, President’s Scholarship, Belt and Road Initiatives Scholarship (BRI), University Scholarships, and many other provincial scholarships. Due to the numerous scholarship opportunities available for international students, China has emerged as one of the favorite destinations for international students in Asia.

The findings revealed that international students who held a Chinese scholarship suffered financial problems when they left China because the Universities had deferred monthly stipends from July 2020. The universities directed that those international students who had left China will not get a monthly stipend. The scholarship will be reimbursed once they return to China and resume their studies. The grant of Chinese scholarship was an essential source of living costs for majority of international students, especially from developing countries. The unavailability of the stipend during the crisis put the international students in a stressful situation. Some participants highlighted that going back to China seems impossible now because they were stuck since 2020; therefore, there was a sense of uncertainty about whether they could get that reimbursement of scholarship money.

“We are suffering more than a COVID-19 patient because our studies are at stake, and also our scholarship is postponed. This is a major problem because I cannot ask my parents for financial support.” (Male, Ph.D. student, Bangladesh)

“I face financial problems because everything is close, and I cannot start part-time work. My monthly stipend is also stopped. This is a huge problem every international student in China is facing. We are not sure we can go back again and receive our pending money” (Male, Ph.D. student, Pakistan)

This section highlights that international students encountered financial problems due to the deferment of their scholarship stipend. The participants expressed that they could not work in their home countries due to the COVID-19 restriction. Therefore, the factor posed an extraordinary impact on international students.

#### 5.2.4. Online learning

The Chinese government implemented emergency regulations such as closing universities and canceling all physical activities soon after the outbreak. However, the learning process was not disturbed with the help of online classes. The Chinese universities shifted the mode of learning to online teaching programs; however, many universities encountered obstacles in online learning procedure, such as a lack of online teaching experience, communication and information gap, and sometimes weak online infrastructure.

The findings reveal that international students who have been stranded in their home countries faced severe problems in online learning. The key hindrances in online classes were, tremendous time zone difference, lack of internet facilities in remote areas, lack of knowledge using Chinese applications, and language barrier. Furthermore, there were massive enrolment of MBBS students in Chinese universities, and they required practical classes to meet the award of degrees. MBBS students were uncertain about finding a job with online learning degrees.

“Online classes are just a waste of time. I miss my online classes at 5 a.m., as per India's standard time. Secondly, my government will not allow me to participate in an internship or medical entry test if I get an online degree. Indian students pursuing MBBS in China are now under immense pressure because they are not able to get back for further studies for one and half years to date” (Male, MBBS student, India)

“We pay high tuition fees for learning, but the problem is online courses. Internet is another problem. For good speed, I need to pay more and sometimes there is no electricity, so everything is just mess” (Female, MBBS student, Nepal)

“At this point, I want China to be straightforward and tell us they will not take us back this year. The uncertainty and glimpses of hope that always turns out to be false are just as mentally damaging as the low-quality online classes” (Male, Ph.D. student, Pakistan)

The theme explicitly describes online learning experiences where international students encountered severe problems such as time zone differences, language barriers, lack of learning, high cost of internet, and dissatisfaction. Therefore, the students were desperate and demanded an immediate return to China to resume their studies.

#### 5.2.5. Challenges to postgraduate and research students

The data revealed that postgraduate and doctoral students who completed the required coursework and were involved in research phase encountered serious challenges. The collected data highlighted that research based international students were struggling to meet their degree requirements in the stipulated time because they required lab experiments and other necessary fieldworks in order to write dissertations. Since the international students were away from China, they were unable to access research activities which caused a serious delay in graduation. Some participants suspended or freeze academic year 2021 due to these unavoidable challenges. The participants expressed that they had no contact with their concerned supervisors and research teams. This factor also hindered academic performance and satisfaction. In addition, many participants were stressed that their visas have been expired and could not return back to school to complete the degrees which was prime objective of the students.

“If I cannot complete my experiments, how can I write a dissertation? Because of the long delay in returning to China, I have suspended my degree duration for one year, from July 2020 to June 2021. However, it is still unclear whether we can return to our schools in September 2021. Our visas already expired in July 2020.” (Male, Ph.D. student, Pakistan)

“I discussed the issue with my supervisor, but she advised me to postpone my studies this year. I have done so, but still no chance to go back. I am afraid I cannot get a degree and waste three years. In short, I am neither here nor there, somewhere in between” (Female, Ph.D. student, Vietnam)

The theme highlights significant challenges faced by postgraduate international students. Due to unavailability of research environment and detachment from labs resulted in severe psychological pressure. The valid visas of the majority of students have been expired and they were in uncertain state of mind whether to return to school or not. The sense of dissatisfaction has compelled many students to suspend or freeze their studies which further resulted in delayed graduation and loss of employment opportunities. These major factors also hindered the readjustment of international students in their home countries.

#### 5.2.6. Campaign

Since May 2020, the pandemic situation gradually normalized in China and a significant reduction was observed in Corona positive cases. Many students intended to raise their voice for an immediate return to their schools in China. The authors observed that international students who were stranded in their home countries were connected with other international students through various communication technologies. These students created many social media online groups with hashtags such as #takeUsBackToChina, and #TakeUsBackToSchool on Twitter. The students also created various online groups in WeChat with the titles “#takeUsBackToChina,” “Mutual Cooperation,” and “Twitter Campaign Team.” Each group contained 500 members (international students) from various countries. Through these online groups, international students from different backgrounds interacted and shared their experiences regarding new updates from Chinese universities, government notices, calls for online protests, and other essential topics related to their experiences. A Thai student tweeted on May 28, 2021:

“Dear all authorities. We are waiting for ‘further notice.’ We have been waiting approximately for the past 16 months, and we cannot stand waiting anymore. I Hope Thai and other international students can return in September 2021 #takeUsBackToChina”

Participants highlighted that they were requesting authorities *via* Twitter to allow vaccinated students to return China following health protocols. An Indian student tweeted:

“Please allow fully vaccinated international students to go back to study in China. Even the quarantine still needed is all right at all. There are many ways to help us. Please have consideration for us. We have always been waiting for your further notice. #takeUsBackToChina”

“It is almost 15 months since we have been attending online classes. This is nothing but just a waste of time. I have wasted 15 months of my life. The vaccine is available, and students are agreed to be quarantined. Still, is it a big deal to call us back? #takeUsBackToChina” (Male, Ph.D. student, Bangladesh)

To sum up, the theme demarcates international students’ desperation to return China and resume studies. In order to highlight their situation to the authorities, international students created online social media groups to raise their voices. Many students recorded protests to allow vaccinated international students with a quarantine policy. The long distance from school put them in a stressful and desperate situation not only affected their academic ventures but also their socio-psychological adjustment and well-being.

## 6. Discussion

Through the lens of mindsponge concept (Vuong and Napier, 2015; Vuong, 2023), the study tried to explain how international students in China absorb new or partially new values and integrate in their mindset after their sudden transition to home countries due to the pandemic. For international students, the acculturation process (culture shock) and re-acculturation process (reverse culture shock) are shifts in mindset. During the COVID-19 pandemic, the shift in international students’ value system was due to information filtering. The process determined the international students’ decision to either accept or reject contemporary values. The process updates international students’ social identity, attitudes, and behaviors (Jin and Wang, 2022). While the goal is to adapt to the current living environment, the decision of whether to accept or reject a certain value is based on individual cost–benefit analysis in specific contexts. Those who has the capacity to integrate new values are more expose to innovative ideas (Napier and Nilsson, 2008). The mechanism offers insights to acculturation and re-acculturation in the context of international students’ literature.

This study explored the experiences of international students in China during the pandemic. An unplanned transition from a normal life to pandemic situation brought various changes in international students’ mindset which impacted their core values which was already held while studying in China. For instance, the participants had spent more than 3 years in China where they successfully adjusted in the host society. After a sudden return to their home culture, they have confronted severe reverse culture shock and loss of group membership (social identity). It also tries to overview the disillusioned challenges international students have faced since the COVID-19 outbreak, which received scant attention in the international literature. The study demonstrates how international students compared perceived differences in their lives in China and back in their homes. The results show that the COVID-19 pandemic presented myriad challenges to international students, such as disruption in the study environment, loss of social networks within and outside China, and readjustment issues at home. The study derived that social identity transition is conceptualized by stressful life events faced by international students, which compromise their well-being since it needs group membership to adjust to the new situation (Jetten et al., 2009; Praharso et al., 2017; Raja et al., 2021).

The experiences of international students were analyzed in two groups. The first group remained in China and witnessed the situation of the pandemic, while the second group had left China during the pandemic and been stranded in their home countries. Comparing these two groups highlights several barriers that changed mindsets of international students in the pandemic context.

In line with the recent study by Yang (2020), international students in China faced severe challenges from December 2020 to May 2021; however, the situation became normal afterwards. They appreciated the efforts of the Chinese government to take necessary measures to minimize the adverse effects of the pandemic (Yang, 2020). Despite the length of their stay, the participants described their experiences in China as being incredibly enlightening where they secured an open mindset to absorbed and integrated new cultural values. Their experience only went adverse after they return to their home countries. The findings revealed that although no participant had finished their studies while in China, they secured strong group membership and social identities and were not particularly ready to return to their former selves and lifestyles they had left behind. The participants expressed that they have evolved and were unable to revert to their former selves.

The results show that the pandemic presented numerous challenges to international students, such as disruption of study environment, loss of social networks, and severe reverse culture shock (Winkel et al., 2021). The study pointed that the shift of mindset of international students is conceptualized by uncertain events faced by international students, which compromises their well-being since it requires group membership to adjust to the new life situation (Jetten et al., 2009, Praharso et al., 2017, Raja et al., 2021).

Since the participants were positively adjusted in China both academically and socially (Raja et al., 2021), the re-transition to home countries was “unplanned,” therefore, students were not mentally ready to adjust and reintegrate into their native culture. The multicultural characteristics and attributes possessed by participants were incompatible with the native cultural values, hence their mindset was not compatible with the new or partially new values which they left behind. As a result, their experiences regarding unplanned return to country of origin provided new insight into shift of mindset debates and re-acculturative stress.

The findings further demonstrated that international students found it challenging to reintegrate in the home society. The findings are in line with previous studies (Xia, 2009; Pritchard, 2011; Kartoshkina, 2015; Yang et al., 2018), that the process of experiencing reverse culture shock on returning to one’s home culture is more problematic and harmful than culture shock. This study also emphasized that the unplanned returning due to a global pandemic is a more serious form of reverse culture shock. The study demarcates that reverse culture shock can have serious and long term repercussions on international students behavior and their concept of self “who am I?” They also experienced social identity loss due to absence of previous group membership in host culture. The participants described the experience of their reverse culture shock as being more distressing, ranging from loss of important group membership to despair and anxiety brought on by a sense of isolation.

Apart from re-acculturative issues, the sudden shift also produced challenges, such as reverse culture shock, loss of interpersonal contacts (Firang, 2020; Xiao, 2020), financial problems (Cao et al., 2020), lack of academic performance (Agius et al., 2021), depression, social withdrawal, making friends, loneliness, visa expiry/graduation delay (Li et al., 2021), problems in online learning (Herman, 2020; Xiong et al., 2020), and sense of belongings (Raja et al., 2021; Wilczewski et al., 2021). The study observed that international students’ mindset has been changed and more open to accept new cultural values while their long stay in host country. Their behavior, attributes and social identities were not the same as before, therefore, they have been struggling to adapt their former selves. This shows that international students have gained positive social identities through diverse group membership in China, therefore, the re-transition to home culture made them struggle to adapt. These findings are in line with previous studies that transition has the capacity to affect group membership (Miller, 2010; Ng et al., 2018; Haslam et al., 2020). The absence of strong group memberships and associated social identities produce serious challenges to individuals’ socio-psychological adjustment (Cruwys et al., 2020).

The findings also revealed positive aspects of international students’ connectedness with their peers through communication technologies during pandemic. They created various online groups to remain connected and share their experiences. These online group membership paved the way for connecting students from many countries on one platform (Raja et al., 2021).

This study tried to unfold these critical insights related to the vulnerable group of international students during the pandemic. The global pandemic presented an ongoing debates for the policymakers including internationalization of higher education agencies worldwide. In addition, international students might carry on online learning from countries but for postgraduate and research students virtual learning may not be more effective. As the internationalization of higher education is deem necessary for many countries including China, there is a dire need for the relevant bodies to devise institutional student support system during such critical phases as the pandemic would have long term consequences. The unplanned transition of international students to home countries during pandemic presented debate about international students’ shift of mindset whether to accept or reject new cultural values and change in their group belongings. The findings from the present study may further guide government and higher educational institutes in preparing to respond to an unexpected crisis in future. The international students’ experiences portray a sense of isolation and dissatisfaction in the difficult phase of life, which can impact the decision of other students before choosing destination for studying abroad.

The study also observed that despite potential challenges, international students expressed increase in their self-confidence. This study has demonstrated the need for improved academic and social support networks for students who have studied abroad when they return home. For students who have recently returned from an extended time of study abroad, there are currently no counseling services available. Policymakers of international educational institutions should concentrate on their reintegration into social and academic systems, which has proven to be a particularly difficult task.

## 7. Conclusion

The mindsponge concept is a helpful notion to explore the experiences of international students during the process of their life transition. This study concluded that the international students’ shift of mindset provides new insights to highlight acculturation and re-acculturation debates during the time of crisis (pandemic). The unplanned return of international students to their home countries without completing their studies remains a subject of great concern in the context of global pandemic. The students with multicultural values found it challenging to reintegrate in home culture without ejecting existing values that was gained while studying abroad. They described effects of reverse culture shock as being very distressing, ranging from loss of group membership to despair and anxiety brought on by a sense of isolation. They perceived their disappointments and frustrations due to loss of previously held social identity and sense of belonging to the traditional society they left behind. There is a need of future studies on the long-term effect of unplanned transition on psychological, social and professional experiences due to reverse culture shock during the global pandemic. The process of re-acculturation has proven to be a challenging endeavor that continued to be a daily challenge. However, from the finding of the study has shown that policymakers in international education should focus on supporting students in future unforeseen crisis. The data and the findings may guide policymakers in higher educational institutes in preparing to respond to an unexpected crisis.

### 7.1. Limitations and possible future research

The study has deepened our understanding of international students’ experiences and challenges in adapting to life changes amid the global pandemic. Along with its strengths, the study has some limitations which may affect the conclusion. Since face-to-face interviews were difficult during the pandemic, the data was collected on a small scale through communication technologies. Future studies with a mixed method may attest to the validity of the process. The participants’ selection was made possible through personal contact and snowballing, as the pandemic caused difficulty in reaching the participants. It might be the case that we have missed giving voice to the experiences of those passing through painful experiences. In addition, most international students were studying on a scholarship basis; therefore, the experiences of self-finance students might be different, which could not be fully captured in the present study. Future research may also look into this aspect. The available data may contribute to the upcoming debates when the pandemic is over. A prospective study may also be conducted to investigate whether the enrolment of international students will decline due to the prevailing crisis.

## Data availability statement

The original contributions presented in the study are included in the article/supplementary material, further inquiries can be directed to the corresponding author.

## Author contributions

RR, JM, and XYL contributed to conception and design of the study. RR and MZ conducted interview scheduled and data collection. MZ, JM, NSA, AHA and XYL performed the analysis. RR wrote the first draft of the manuscript. JM, and MZ wrote sections of the manuscript. NSA and AHA contributed in revising the theoretical framework of the article. All authors contributed to manuscript revision, read, and approved the submitted version.

## Funding

Funded by Pakistan Studies Centre at North Minzu University, Yinchuan, Ningxia, China, (2023GBYJ-001).

## Conflict of interest

The authors declare that the research was conducted in the absence of any commercial or financial relationships that could be construed as a potential conflict of interest.

## Publisher’s note

All claims expressed in this article are solely those of the authors and do not necessarily represent those of their affiliated organizations, or those of the publisher, the editors and the reviewers. Any product that may be evaluated in this article, or claim that may be made by its manufacturer, is not guaranteed or endorsed by the publisher.
